# In Chagas disease, transforming growth factor beta neutralization reduces *Trypanosoma cruzi* infection and improves cardiac performance

**DOI:** 10.3389/fcimb.2022.1017040

**Published:** 2022-11-30

**Authors:** Roberto Rodrigues Ferreira, Elen Mello de Souza, Glaucia Vilar-Pereira, Wim M. S. Degrave, Rayane da Silva Abreu, Marcelo Meuser-Batista, Nilma Valéria Caldeira Ferreira, Steve Ledbeter, Robert H. Barker, Sabine Bailly, Jean-Jacques Feige, Joseli Lannes-Vieira, Tania C. de Araújo-Jorge, Mariana Caldas Waghabi

**Affiliations:** ^1^ Laboratório de Genômica Funcional e Bioinformática, Instituto Oswaldo Cruz, Fundação Oswaldo Cruz (Fiocruz), Rio de Janeiro, Brazil; ^2^ Laboratório de Inovações em Terapias, Ensino e Bioprodutos, Instituto Oswaldo Cruz, Fundação Oswaldo Cruz (Fiocruz), Rio de Janeiro, Brazil; ^3^ Laboratório de Virologia Molecular, Instituto Oswaldo Cruz, Fundação Oswaldo Cruz (Fiocruz), Rio de Janeiro, Brazil; ^4^ Laboratório de Biologia das Interações, Instituto Oswaldo Cruz, Fundação Oswaldo Cruz (Fiocruz), Rio de Janeiro, Brazil; ^5^ Departamento de Anatomia Patológica e Citopatologia, Instituto Nacional de Saúde da Mulher, da Criança e do Adolescente Fernandes Figueira, Fundação Oswaldo Cruz (Fiocruz), Rio de Janeiro, Brazil; ^6^ Tissue Protection and Repair, Sanofi-Genzyme R&D Center, Framingham, MA, United States; ^7^ Laboratory BioSanté, Université Grenoble Alpes, INSERM, CEA, Grenoble, France

**Keywords:** cardiac fibrosis, Chagas disease, TGF-β, 1D11, treatment

## Abstract

Chronic Chagasic cardiomyopathy (CCC), a progressive inflammatory and fibrosing disease, is the most prominent clinical form of Chagas disease, a neglected tropical disease caused by *Trypanosoma cruzi* infection. During CCC, the parasite remains inside the cardiac cells, leading to tissue damage, involving extensive inflammatory response and irregular fibrosis. Among the fibrogenic factors is transforming growth factor-β (TGF-β), a key cytokine controlling extracellular matrix synthesis and degradation. TGF-β is involved in CCC onset and progression, with increased serum levels and activation of its signaling pathways in the cardiac tissue, which crucially contributes to fibrosis. Inhibition of the TGF-β signaling pathway attenuates *T. cruzi* infection and prevents cardiac damage in an experimental model of acute Chagas disease. The aim of this study was to investigate the effect of TGF-β neutralization on *T. cruzi* infection in both *in vitro* and *in vivo* pre-clinical models, using the 1D11 monoclonal antibody. To this end, primary cultures of cardiac cells were infected with *T. cruzi* trypomastigote forms and treated with 1D11. For *in vivo* studies, 1D11 was administered in different schemes for acute and chronic phase models (Swiss mice infected with 10^4^ parasites from the Y strain and C57BL/6 mice infected with 10^2^ parasites from the Colombian strain, respectively). Here we show that the addition of 1D11 to cardiac cells greatly reduces cardiomyocyte invasion by *T. cruzi* and the number of parasites per infected cell. In both acute and chronic experimental models, *T. cruzi* infection altered the electrical conduction, decreasing the heart rate, increasing the PR interval and the P wave duration. The treatment with 1D11 reduced cardiac fibrosis and reversed electrical abnormalities improving cardiac performance. Taken together, these data further support the major role of the TGF-β signaling pathways in *T. cruzi*-infection and their biological consequences on parasite/host interactions. The therapeutic effects of the 1D11 antibody are promising and suggest a new possibility to treat cardiac fibrosis in the chronic phase of Chagas’ heart disease by TGF-β neutralization.

## Introduction

Chagas disease, also known as American trypanosomiasis, is caused by the flagellate protozoan *Trypanosoma cruzi*, transmitted by a triatomine insect vector, commonly known as kissing bugs (World Helth Organization, 2021). Chagas disease presents distinct and successive acute and chronic phases. In humans, signals of the acute phase begin 6 to 10 days after infection and last for about 4 to 8 weeks (Steverding, 2014). In most cases, the acute phase is hardly identified with the absence of symptoms or by presenting nonspecific clinical symptoms, such as fever and malaise (Chagas, 1909; Dias, 1984; Rassi et al., 2008). If not treated or in case of treatment failure, the acute phase progresses into the chronic phase (Marin-neto et al., 2002; Coura, 2007). Chronic chagasic cardiomyopathy (CCC) is the main and most severe clinical manifestation of chronic Chagas disease, representing an important problem in terms of public health (Álvarez et al, 2014) and social impact (Amieva et al., 2021). In the affected individuals, alterations in heart function such as arrhythmias, apical aneurysms, thromboembolism and progressive heart failure may result in sudden death (Rassi et al., 2010). CCC is also the most common cause of non-ischemic cardiomyopathy in Latin America, representing approximately 10,000 deaths/year in patients between 30 and 50 years old, in endemic areas of Chagas disease (Rassi et al., 2010; Bocchi et al., 2017).

In the chronic phase of infection the parasite remains in myocardial cells contributing to the damage in cardiac tissue, which involves inflammatory response, cell death, and focal fibrosis (Sato et al., 1993; Andrade, 1999; Rassi et al., 2012). Mononuclear cells-enriched cardiac inflammation is directly related to the intensity of myocardial fibrosis, a biological process conducted by cytokines and growth factors that act on fibroblasts and stimulate collagen production (Rossi, 1998; Pereira et al., 2014; Pereira et al., 2015). This damage leads to structural modifications involving tissue repair and remodeling, and results in replacement of cardiac tissue with connective tissue, characteristic of the fibrotic process (Ihn, 2002; Zamilpa et al, 2014). In view of the importance of fibrosis in cardiac involvement during CCC, mechanisms involved in this process represent potential targets for therapeutic strategies to prevent or reverse fibrosis to improve the prognosis of patients with CCC, since there are still no specific treatments for this phase of the disease. The molecule that stands out as a key regulator in the production and remodeling of the extracellular matrix in fibrosis is TGF-β.

TGF-β is a multifunctional cytokine, which plays a role in regulating processes such as cell apoptosis, embryogenesis, epithelial cell migration and epithelial-mesenchymal transitions (Moustakas and Heldin, 2005). In mammals, there are three isoforms of TGF-β: TGF-β1, TGF-β2 and TGF-β3. These isoforms share homology of about 70% in the amino acid sequence and are encoded by distinct genes located in different chromosomes (Huang et al., 2014). However, TGF-β main biological activities are associated with the regulation of cell proliferation and differentiation, immunosuppressive activities, and regulation of the deposition of extracellular matrix components (Reisdorf et al., 2001; Moustakas and Heldin, 2005). Several studies have reported the involvement of TGF-β in cardiac fibrosis (Eghbali et al., 1991; Okada et al., 2005; Tan et al., 2010; Bhandary et al., 2018; Frangogiannis, 2020; Ko et al., 2022) and in other organs such as kidney, liver and lung (Daniels et al., 2004; Wang et al., 2005; Gellibert et al., 2006; Petersen et al., 2008; Dewidar et al., 2019; Gu et al., 2020; Inui et al., 2021).

In the last two decades, our group has been studying the role of TGF-β as an inducer of several pathological processes in Chagas disease (Ferreira et al., 2022; Waghabi et al., 2022). We already investigated TGF-β pathway inhibition in experimental models of acute and chronic Chagas heart disease using SB431542 and GW788388, which are two selective inhibitors of the kinase activity of the TGF-β type 1 receptor TβR1/ALK5 (Waghabi et al., 2007; de Oliveira et al., 2012; Ferreira et al., 2019). The treatment with TGF-β pathway inhibitors improved cardiac parameters as it (i) reduced the prolonged PR and QTc intervals, increased heart rate, and reversed sinus arrhythmia, and atrial and atrioventricular conduction disorders; (ii) reversed the loss of connexin-43 enriched intercellular plaques and fibrosis of the cardiac tissue; (iii) reduced activation and expression of TGF-β intracellular proteins (Smad2/3); (iv) modulated protein expression of fibrosis regulators (MMP-9, reduced TIMP-1/TIMP-2/TIMP-4); and (v) partially restored GATA-6 and Tbox-5 transcription, supporting cardiac recovery (Ferreira et al., 2019). Understanding the mechanisms triggered by the strategies inhibiting TGF-β activity and the interference of these factors in the development of Chagas’ cardiopathy are of great importance. It makes TGF-β a relevant target for the development of therapies for chronic heart disease patients. However, the clinical use of inhibitors of TGF-β receptor kinase activity could induce some deleterious secondary effects, such as heart-valve lesions, physeal dysplasia and an increase in femur and tibia thickness (Anderton et al., 2011). Thus, investigating new compounds that inhibit the TGF-β pathways by other mechanisms is of utmost importance. In the present study, we used *in vitro* and pre-clinical models to investigate the effect of a neutralizing antibody (1D11), directed against the three TGF-β isoforms, on *T. cruzi* infection.

## Methods

### Ethics statement

All mice procedures were carried out in strict accordance with the recommendations in the Guide for the Care and Use of Laboratory Animals of the Brazilian National Council of Animal Experimentation (http://www.cobea.org.br/ ) and the federal law 11.794 (8 October 2008). Protocols used in this study were approved by the Institutional Committee for Animal Ethics of Fiocruz (CEUA/Fiocruz, Licenses LW10/14 and LW42-11). All efforts were made to minimize animal suffering.

### Parasites

Trypomastigotes of the Y strain of *T. cruzi* were obtained from the blood of infected mice at the peak of parasitemia (Meirelles et al., 1986) and were maintained in serum-free medium with 2% bovine serum albumin.

The Colombian *T. cruzi* DTU I strain (Zingales et al., 2012) was maintained by serial passages in specific pathogen-free C57BL/6 mice by IP injection of 5,000 blood trypomastigotes in 0.2 mL of pyrogen-free saline (BioManguinhos-Fiocruz, Brazil) every 35 days post-infection (dpi), the parasitemia peak using this inoculum.

### Primary cultures of cardiomyocytes

Cardiac cells from mouse embryos were obtained, and as previously described these cells were contaminated with few fibroblasts and endothelial cells (Meirelles et al., 1986). Cells were maintained in Eagle’s medium (Sigma) supplemented with 7% fetal calf serum (FCS) (Sigma), 100 µg/ml gentamicin (Sigma), 1 mM L-glutamine (Sigma), and 2.5 mM CaCl_2_.

### Drug

1D11 is a murine IgG1 monoclonal antibody that neutralizes all three TGF-β isoforms. The 1D11 antibody and its isotype matched murine IgG1 control (13C4) were produced by Genzyme Corporation (Sanofi-Genzyme, USA). The vehicle used for these antibodies was composed of 20 mM histidine, 135 mM NaCl, 10 mM methionine, 0.01% tween 80.

### Infection and treatment in *in vitro* assays

Cardiac cells were seeded over gelatin-coated glass coverslips in 24-well plates (1 x 10^5^ cells/well) for 24 h at 37°C under an atmosphere of 5% CO_2_. Cultures were incubated with fresh medium containing 25, 100 and 200 µg/mL 1D11 and 100 µg/mL 13C4 or vehicle for 2 h before infection or during the addition of trypomastigotes of the Y strain in a parasite-to-host cell proportion of 10:1. Parasites were also pre-treated with 100 µg/mL 1D11 for 2 h before infection. At the time indicated, cells were washed with phosphate-buffered saline (PBS), fixed in Bouin’s solution, and stained with Giemsa solution. The percentage of cardiac cells containing parasites and the number of parasites per infected cell were determined by counting 400 cells/slide on two distinct coverslips at 24, 48, 72, and 96 h post infection. Analysis was performed with a Zeiss microscope at a magnification of x400. Data are means ± standard deviations from three independent experiments.

### Infection and treatment in *in vivo* assays

#### Acute phase model

Male Swiss mice (age 6 to 8 weeks, weight 18 to 20 g) were obtained from the animal facilities of ICTB (Instituto de Ciência e Tecnologia em Biomodelos (ICTB/FIOCRUZ, Rio de Janeiro, Brazil). Mice were housed for at least 1 week before parasite infection under environmental factors and sanitation according to the “Guide for the Care and Use of Laboratory Animals”. Infection was performed by intraperitoneal (IP) injection of 10^4^ bloodstream trypomastigotes from the Y strain of *T. cruzi*. Sex and age-matched noninfected mice were maintained under identical conditions.

#### Chronic phase model

Female C57BL/6 mice (H-2^b^, aged 4 to 6 weeks) were obtained from the animal facilities of ICTB (FIOCRUZ, Rio de Janeiro, Brazil). Animals were housed for at least one week before parasite infection at Animal Facility/IOC under environmental factors and sanitation according to “Guide for the Care and Use of Laboratory Animals”. For all experimental procedures, C57BL/6 mice were infected by IP injection of 100 blood trypomastigotes of the Colombian strain of *T. cruzi* (Ferreira et al., 2019). Parasitemia was employed as a parameter to establish acute and chronic phases using 5 μL of blood obtained from the tail vein and it was also individually checked by direct microscopic counting of parasites, as previously described (Brener, 1962; Waghabi et al., 2009).

#### 1D11 treatment

Treatment was performed by intraperitoneal (IP) administration (0.2 mL) of the 1D11 antibody, and its isotype matched murine IgG1 control (13C4). For determination of the best dose, 1D11 was IP administered to Y-infected mice at 5 dpi with 1.25, 2.5, 5 and 10 mg/Kg. Further, as the best-chosen dose, mice received 5mg/Kg 1D11 in different schemes of treatment for acute and for chronic phase assays. The control group received vehicle buffer using the same scheme.

#### Experimental groups

Mice were divided into the following groups respecting the limit of 5 animals per cage: untreated and non-infected (NI), untreated and *T. cruzi* infected (*T. cruzi*) and 1D11 treated and *T. cruzi* infected, using 5 treatment schemes for the acute phase studies: single dose at 3 dpi (SD d3), single dose at 5 dpi (SD d5), day 0+ three times a week (d0+ 3xw), day 3+ once a week (d3+ 1xw), and day 3+ three times a week (d3+ 3xw). For chronic phase studies, mice were divided into the following groups: untreated and non-infected (NI), untreated and *T. cruzi* infected (*T. cruzi*) and 1D11 treated and *T. cruzi* infected, using 2 treatment schemes: single dose at 120 dpi (SD d120) and day 120+ once a week (d120+ 1xw). Eight to 10 mice from each group were used for analysis at each different dpi, and three independent experiments were performed to assure reproducibility.

#### Mortality and parasitemia

The mortality of the mice was checked daily until 30 dpi (for acute phase model) and until 150 dpi (for chronic phase model) and expressed as a percentage of cumulative mortality. To check the effects of treatments on parasite circulation, parasitemia was individually checked by direct microscopic counting of parasites in 5 µl of blood, as described above.

#### Electrocardiography analysis

ECG recording and analysis were performed in all groups of infected and non-infected animals. Mice were IP tranquilized with diazepam (20 mg/Kg), fixed in the supine position and the transducers were carefully placed subcutaneously according to chosen preferential derivation (DII). Traces were recorded using a digital system (Power Lab 2/20) connected to a bio-amplifier at 2 mV for 1 s (PanLab Instruments, Spain). Filters were standardized between 0.1 and 100 Hz and traces were analyzed using the Scope software for Windows V3.6.10 (PanLab Instruments, Barcelona, Spain). ECG parameters were recorded for at least 2 min and evaluated in the acute (at 15 dpi) and chronic phases (at 120, 150 and 180 dpi), using the following standard criteria: the heart rate, monitored by beats/minute (bpm), and the variation at P wave and PR, QRS and correct QT intervals (QTc), all measured in milliseconds (ms). The ECG parameters were analyzed as previously described (Ferreira et al., 2019).

#### Histological assessment of cardiac fibrosis

Formalin-fixed tissues were dehydrated and embedded in paraffin. Sections (3 μm) were stained by Masson’s trichrome as previously described (Ferreira et al., 2019). Sections were observed using a Nikon microscope coupled with image acquisition systems (Nikon) and the images were assessed for percentage area of collagen using CellProfiler image analysis software (http://www.cellprofiler.org). In addition, percent of Masson’s Trichrome stained area (light blue areas) was quantified for each heart section in a 10x microscopic magnification. Two heart sections were made for each mouse, and 4-6 fields were quantified per section to obtain whole tissue information.

#### Statistical analysis

Differences between infected and non-infected groups and between infected mice, 1D11 treated or not, were considered statistically significant when *p< 0.05, **p< 0.01, and ***p< 0.001, as determined by GraphPad Prism 8.0 software (GraphPad Software Inc., San Diego, CA, USA). All the analyses were performed using the non-parametric Mann–Whitney test.

## Results

### 1D11 treatment decreases *T. cruzi* invasion of cardiac cells *in vitro*

**Figure 1:**
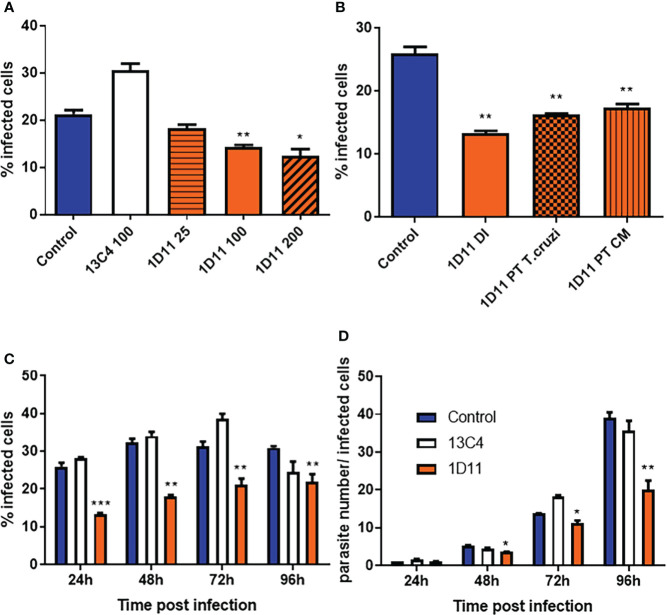
1D11 treatment decreases *T. cruzi* invasion of cardiomyocytes and the parasite number per infected cells. The percentage of cardiac cells containing parasites **(A–C)** and the number of parasites per infected cell **(D)** were determined by counting 400 cells/slide on two distinct coverslips at 24, 48, 72, and 96 h post infection. Data are means ± standard deviations from three independent experiments. Significant differences between infected cells treated or not with 1D11 are indicated by *p < 0.05 **p< 0.01, ***p< 0.001.

Given that it was previously demonstrated that TGF-β and its receptors are required for *T. cruzi* entry into cardiomyocytes (Ming et al., 1995; Waghabi et al, 2005; Waghabi et al., 2007), we first tested the use of 1D11 during the process of invasion of these cells by *T. cruzi*. To this end, cardiac cells were treated with 25, 100 and 200 µg/ml 1D11 for 24 h during *T. cruzi* Y strain infection. **Figure 1A** demonstrates that the addition of 100 and 200 µg/ml 1D11 reduced *T. cruzi* host cell infection. We also tested the pre-treatment of both parasites (TC PT) and cardiac cells (CM PT) with 100 µg/ml 1D11 (**Figure 1B**) and observed similar effect as compared to 1D11 addition during infection (DI). Thus, we chose to use 100 µg/ml 1D11 in the next steps to continue the investigation of TGF-β neutralization during *in vitro T. cruzi* infection on cardiac cells. We performed a kinetic study from 24 h until 96 h of *T. cruzi* infection, and observed a sustained lower percentage of *T. cruzi* infected cardiac cells after the inhibition of TGF-β even after 96 h post infection (**Figure 1C**). Quantification of the percentage of infected cells is shown in **Figures 1A–C**

### 1D11 treatment inhibits the intracellular *T. cruzi* cell cycle

We have previously demonstrated that TGF-β is implicated in *T. cruzi* infection and controls its intracellular life cycle (Waghabi et al., 2005, 2007), thus we examined whether the TGF-β neutralization with 1D11 during the process of *T. cruzi* infection could affect the intracellular parasite cycle. For this purpose, cells were treated with 100 µg/ml 1D11, and compared with controls. Our results showed that the addition of 1D11 reduced the number of intracellular forms of the parasite (**Figure 1D**). Quantification of the number of intracellular parasites during the cycle showed that there was already a significant difference in the mean number of intracellular parasite forms, at 48 h post infection. This difference increased throughout the infection, reaching the highest differences, at 96 h (**Figure 1D**).

### Comparison of the different schemes of treatment with 1D11 *in vivo*

In the second set of experiments, 5mg/Kg antibody was administered *in vivo* by IP injection in male Swiss mice infected with 10^4^ bloodstream trypomastigotes of the Y strain in the acute model. Evaluating parasitemia and survival rates, we observed that treatment starting at 3 dpi (SD d3) was not beneficial to infected mice, resulting in similar parasitemia peak and mortality as compared to untreated group (*T. cruzi*). On the other hand, a single dose of 1D11 treatment at 5dpi (SD d5) showed best results in decreasing the parasitemia peak and protecting mice from death (**Supplemental Figures 1A–C**). Thus, for the subsequent studies, the treatment scheme of 1D11 d5 was chosen. The next step was to test the best dose of 1D11 to be IP administered, and to this end we performed a dose-response assay with 1.25, 2.5, 5 and 10 mg/Kg of 1D11. The results showed a dose-dependent inhibition of parasitemia at 8 dpi from 1.25 up to 10 mg/Kg of 1D11 (**Supplemental Figures 1D–F**) and 5mg/Kg 1D11 d5 was considered as the best dose as it presented similar results as compared to the 10mg/Kg dose (**Supplemental Figures 1D–F**).

### 1D11 treatment reduced parasitemia and increased mice survival rates in acutely *T. cruzi-*infected mice

**Figure 2:**
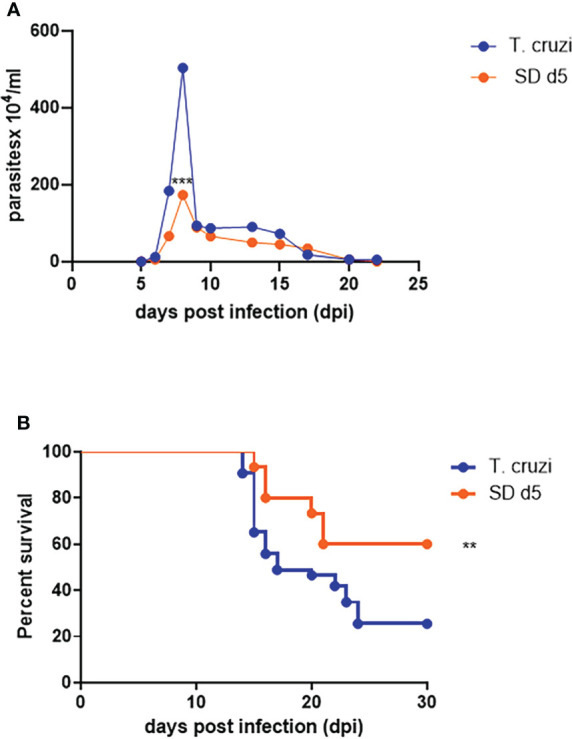
1D11 best dose administration in a single dose scheme at 5 dpi reduced the peak of parasitemia and improved the survival rates. The treatment reduced **(A)** the peak of parasitemia and **(B)** mortality rates after 30 dpi. Data are means ± standard deviations from three independent experiments. Significant differences between infected mice treated or not with 1D11 are indicated by **p< 0.01, ***p< 0.001.

All assays were thus performed with the 5 mg/Kg dose of 1D11 in a single dose scheme at 5 dpi. Under these conditions, 1D11 led to a significant reduction at the peak of parasitemia, reaching 60% of reduction on circulating parasites and 65% of reduction on mortality rates of the mice after 30 dpi (**Figures 2A, B**).

### 1D11 treatment prevented heart damage during acute phase of *T. cruzi* infection

**Figure 3:**
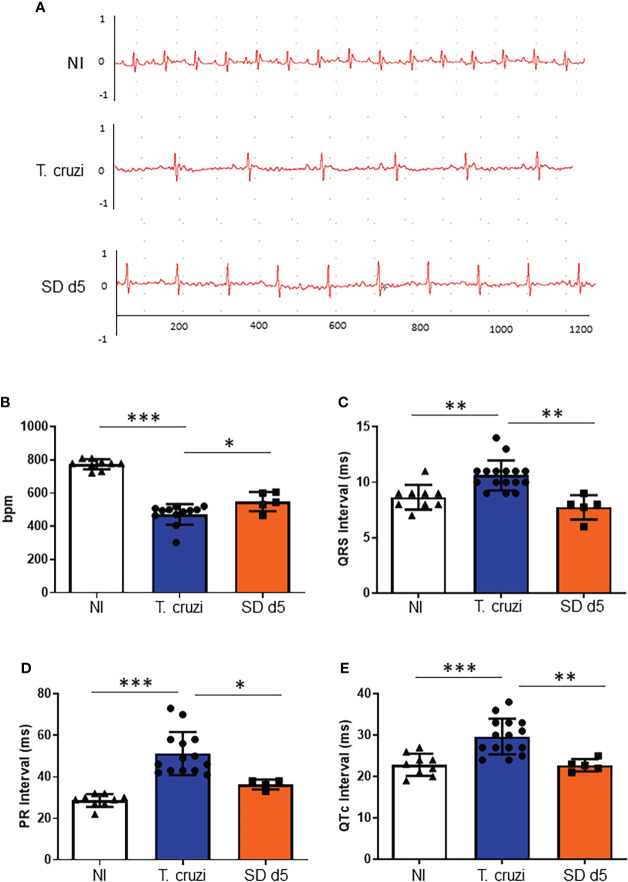
1D11 IP administration restored electrocardiographic parameters. 1D11 was administered at 5 dpi by intraperitoneal injection in male Swiss mice infected with 10^4^ bloodstream trypomastigotes of the Y strain. Representative electrocardiographic tracings of each group at 15 dpi are shown **(A)**. 1D11 treatment resulted in best heart rate, in beats per minute (bpm) **(B)**; PR interval in milliseconds **(C)**; QRS interval **(D)** and QTc interval in milliseconds **(E)**. Significant differences between non infected and infected mice treated or not with 1D11 are indicated by *p< 0.05 **p< 0.01, ***p< 0.001.

At 15 dpi, ECG parameters were evaluated, and the analysis of the ECG traces demonstrated atrial ventricular block with PR interval higher than 40 ms, leading to sinus bradycardia in sham-treated *T. cruzi-* infected mice as compared to the non-infected control group (472.8 and 774.2 bpm, respectively, **Figure 3A**). ECG analysis demonstrated that at 15 dpi, all mice presented a significant decrease in heart rate, as measured by beats per minute (bpm) (**Figure 3B**), associated with a significant increase of QRS complex (**Figure 3C**), PR (**Figure 3D**) and QTc (**Figure 3E**) intervals, when compared with sex- and age-matched non-infected (NI) controls. 1D11 IP administration significantly limited the bpm decrease at 15 dpi, with a mean heart rate of 549.2 bpm (**Figure 3B**). Further, improved QRS complex, PR and QTc intervals were observed in presence of 1D11 (**Figures 3C–E**).

### 1D11 treatment increased mice survival rates in chronically *T. cruzi-*infected mice

**Figure 4:**
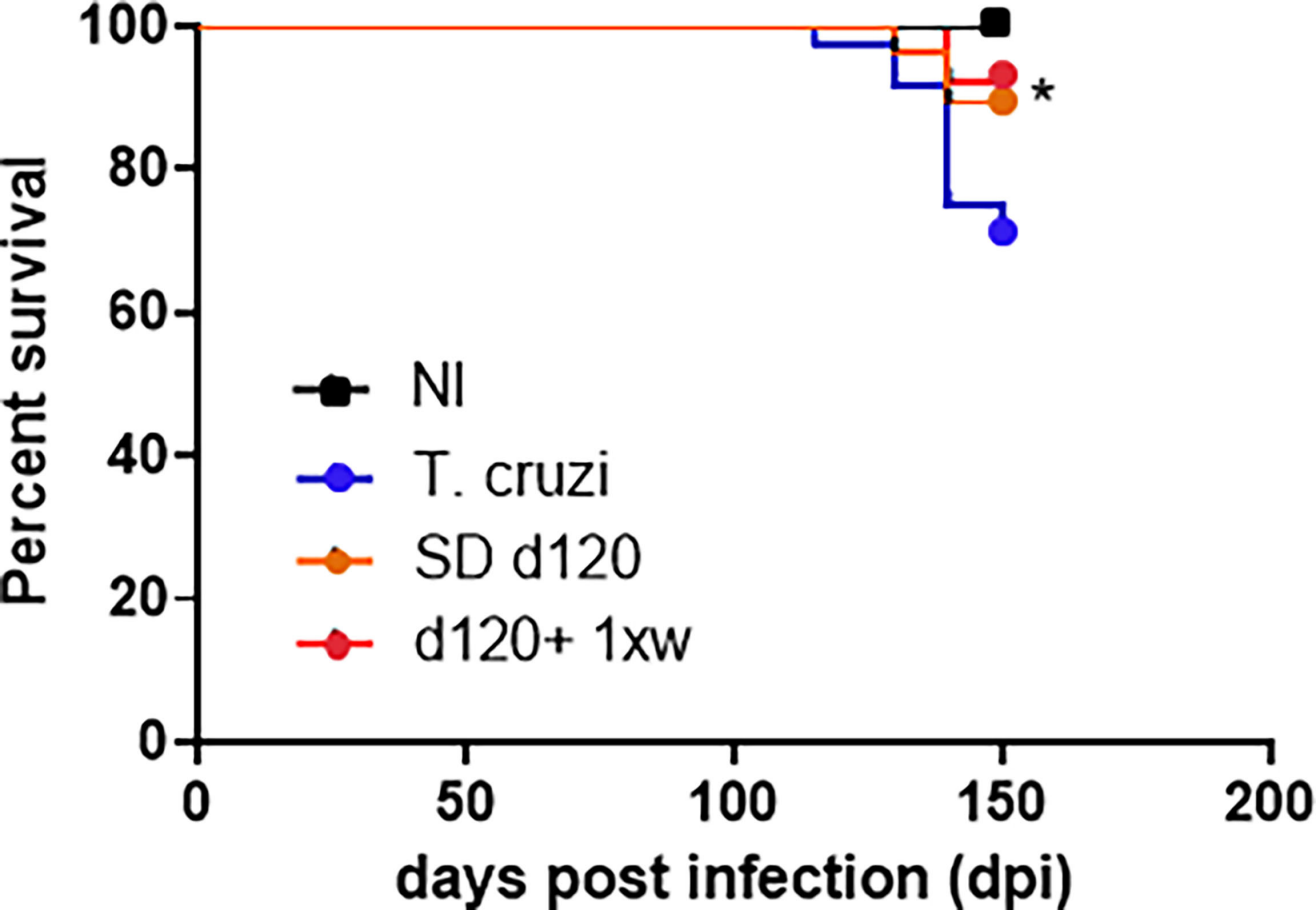
1D11 treatment increased mice survival rates in chronically *T. cruzi-*infected mice. After 120 dpi 1D11 was administered in two schemes: single dose at 120 dpi (SD d120) and starting at 120 dpi + once a week (d120+ 1xw). Significant differences between non infected and infected mice treated or not with 1D11 are indicated by *p< 0.05.

After characterizing the successful effect of a single IP dose of 1D11 during the experimental acute phase of Chagas disease, we aimed to test the possible benefits of such administration in chronic *T. cruzi* infected mice, in which the fibrotic feature is already installed in mice hearts. In the chronic model assay, *T. cruzi*-infected animals presented a circulating parasite peak at 42 dpi. This evaluation was an important step for the confirmation that the animals were infected and would develop Chagas disease (data not shown), and thus, only mice presenting parasites at the parasitemia peak were considered for chronic infection evaluations. Given that best results of 1D11 treatment during the acute phase were obtained with the single dose scheme, we decided to sustain this scheme during the chronic phase and also included a group of mice treated once a week for 30 days starting at 120 dpi, when cardiac damage has already been installed. About 90% of the infected animals survived the acute phase and progressed to the chronic phase of Chagas disease (120 dpi). When treated with 1D11 in both schemes (SD d120 and d120+ 1xw), we observed an increase of approximately 15% in survival of these animals at 150 dpi (**Figure 4**). We did not detect significant variation in body weight between studied groups. Weighing of the heart, liver, kidney, and spleen was also performed, and a significant increase was observed in liver and spleen weight of infected animals compared to the control group, but the 1D11 treatments was not able to act on these alterations (data not shown).

### 1D11 treatment reversed cardiac altered electrical conduction in *T. cruzi-*infected mice models

**Figure 5:**
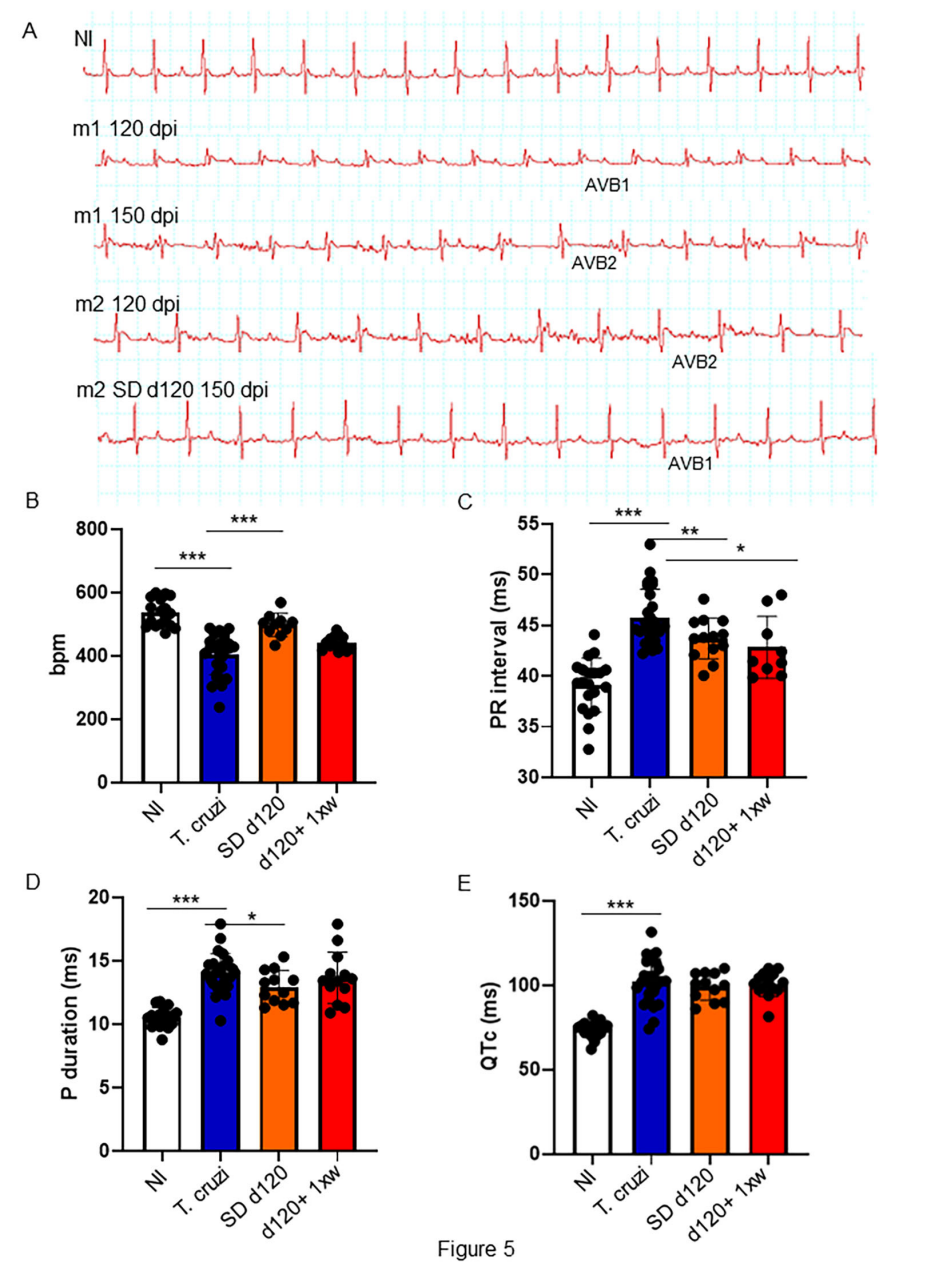
1D11 reverses ECG abnormalities in a mouse model of chronic Chagas’ heart disease. Representative electrocardiographic tracings of each group at 150 dpi are shown **(A)**. 1D11 SD d120 treatment resulted in best heart rate, in beats per minute (bpm) **(B)**; PR interval in milliseconds **(C)**; P duration in milliseconds **(D)** and QTc interval in milliseconds **(E)**. Significant differences between non infected and infected mice treated or not with 1D11 are indicated by *p< 0.05, **p< 0.01, ***p< 0.001.

We further tested the effects of 1D11 administration in animals chronically infected with *T. cruzi* focusing on the heart parameters. To this end, we performed electrocardiogram analysis at the end of the experiment at 150 dpi. Electropherograms were selected to demonstrate the evolution of cardiac electrical alterations observed in the same mouse; thus, an electropherogram of a mouse in 120 dpi (m1 120 dpi) indicates the presence of atrium-ventricular blockage grade 1 (AVB1), which clearly evolved into an atrium-ventricular blockage grade 2 (AVB2) in 150 dpi (m1 150 dpi). After 1D11 administration in single dose, mice improved the heart electrical disorders; in **Figure 5**, we showed a mouse ECG tracing before starting 1D11 treatment at 120 dpi (m2 120 dpi) with AVB2, and after 1D11 SD d120 treatment an AVB1 profile was observed (m2 SD d120 150 dpi) indicating an improved cardiac electrical function. Moreover, we observed that best results were obtained by treatment with 1D11 in a single dose (SD d120) as compared to the group which received treatment once a week for 30 days after 120 dpi (d120+ 1xw) (**Figures 5, D**). The 1D11 single dose scheme increased heart rate parameters (*T. cruzi* = 405.4 ± 64.3/SD d120 = 507.2 ± 43.8; **Figure 5B**); decreased PR interval (*T. cruzi* = 45.7 ± 2.8/SD d120 = 42.2 ± 2.5; **Figure 5C**) and P wave duration (*T. cruzi* = 14.1± 1.5/SD d120 = 13.0 ± 1.1; **Figure 5D**). Mice treated with 1D11 d120+ 1xw just improved PR interval (*T. cruzi* = 45.7 ± 2.8/d120+ 1xw = 43.7 ± 2; **Figure 5C**) with no effect on other parameters. TGF-β neutralization by 1D11 administration had no effect on the prolonged QTc observed in mice chronically infected by *T. cruzi* (**Figure 5D**).

### 1D11 treatment restored collagen deposition in chronically *T. cruzi*-infected mice model

**Figure 6:**
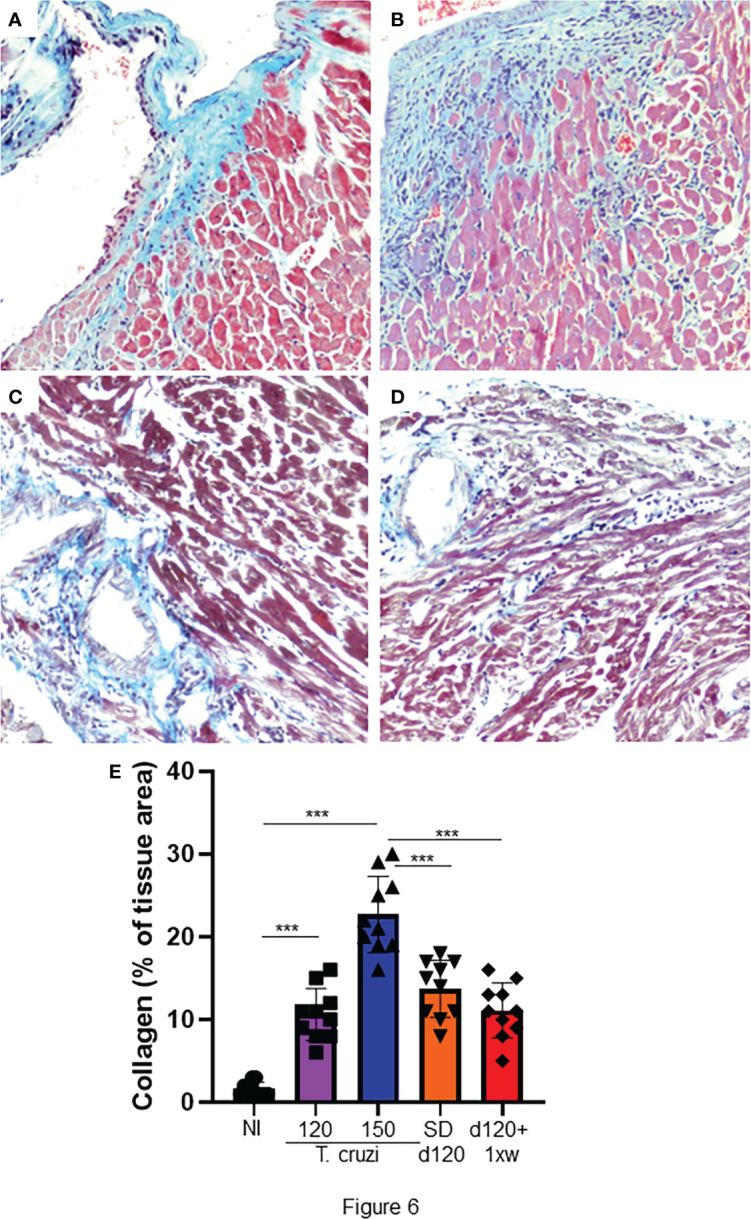
1D11 treatment reverses heart fibrosis in mice chronically infected with T. cruzi. After 120 dpi 1D11 was administered in two schemes: **(C)** a single dose at 120 dpi (SD d120) and **(D)** starting at 120 dpi + once a week (d120+ 1xw). **(A)** Non-infected (NI) and **(B)** infected mice were sacrificed at 150 dpi for fibrosis assessment. Heart sections were stained for collagen deposition by Masson’s trichrome (light blue staining). **(E)** Percent of Masson’s Trichrome stained area (light blue areas) was quantified on microscopic images of heart sections using CellProfiler image analysis software. Data is a representative image of 10 independent mice per group. Significant differences between non infected and infected mice treated or not with 1D11 are indicated by ***p < 0.001.

The most important histopathological finding in CCC is cardiac fibrosis, both in humans and in experimental models (Rossi, 1998). Thus, we assessed whether treatment with 1D11 could reverse heart fibrosis. As already demonstrated in this model, we identified a significant increase in collagen deposition in the heart of animals with 120 and 150 dpi (**Figures 6A, B**, respectively). Both 1D11 treatment schemes decreased heart fibrosis, but we still observed a higher percent of area of collagen deposition in the heart of infected animals as compared to non-infected ones (**Figure 6C–E**). Although 1D11 d120+ 1xw treated mice presented a slight reduction in the collagen area as compared to the group of mice treated in a single dose scheme, SD d120, it was not statistically significant (**Figure 6C–E**).

## Discussion

Even more than a century after its discovery, many challenges remain unresolved when the subject is Chagas disease. Therapeutic and prognostic methods must still be improved (Vilar-Pereira et al., 2016; Pérez-Molina and Molina, 2018). Here, we aimed to investigate the role of TGF-β on *T. cruzi* infection using the 1D11 monoclonal antibody, which neutralizes the three isoforms of TGF-β in *in vitro* and pre-clinical models. We showed that addition of 1D11 to cardiac cells reduces cardiomyocyte invasion by *T. cruzi* and the number of parasites per infected cell. In both acute and chronic experimental models, *T. cruzi* infection altered the electrical conduction: decreasing the heart rate, increasing the PR interval and the P wave duration. The treatment with 1D11 reversed electrical abnormalities, improved cardiac performance, and reduced cardiac fibrosis.

In 2007, we demonstrated that SB-431542, an inhibitor of ALK5, inhibited *T. cruzi*-induced activation of the TGF-β pathway in epithelial cells and in cardiomyocytes. We also described that addition of SB-431542 reduced cardiomyocyte invasion by *T. cruzi*. Furthermore, we concluded that SB-431542 treatment significantly reduced the number of parasites per infected cell and of trypomastigote differentiation and release. To confirm these data we first tested a neutralizing antibody that recognizes human TGF-β 1, 2 and 3 (1D11, Genzyme) on infected cardiac cells from mouse embryos treated with different concentrations of the antibody. The addition of 1D11 reproduced our previous findings: (1) it inhibited cardiac cells invasion by *T. cruzi*, (2) it reduced the percentage of cells infected with *T. cruzi*; and (3) it reduced the number of parasites per infected cell. These results are important, since they demonstrate that neutralizing TGF-β activity before its binding to cell receptors not only inhibits *T. cruzi* invasion but also acts through inhibiting parasite proliferation probably impairing the infection of neighbor’s cells.

Fibrosis is one of the most significant histopathologic findings of CCC, being associated with the presence of inflammatory infiltrates and degeneration of cardiac cells (Rossi and Bestetti, 1995; Rossi, 2001). The mechanisms driving the progression of fibrosis in different organs and tissues are similar and have common features. The progressive accumulation of connective tissue and excessive deposition of extracellular matrix components result in the replacement of normal tissue architecture and loss of functional activity. This process is mediated by soluble cytokines and growth factors, which regulate cell migration, proliferation, and differentiation, as well as the synthesis and degradation of extracellular matrix components, amongst which TGF-β (Gharee-Kermani and Pham, 2001; Hata and Chen, 2016).

In the present study, 1D11 modulated parasitemia and survival rates. Moreover, TGF-β neutralization also improved the cardiac function, when compared with the control group, corroborating other data in the literature. A previous study performed by our group showed that GW788388, a potent selective inhibitor of ALK5, administrated in an acute model of Chagas disease significantly decreased parasitemia, and improved cardiac electrical conduction as measured by PR interval in electrocardiography, restored connexin43 expression, increased survival and decreased cardiac fibrosis. Here, we also showed that TGF-β neutralization starting at 5 dpi exerts beneficial actions through inhibiting the progression of heart commitment by electrical disorders, such as arrythmias.

Recently we investigated TGF-β pathway inhibition by GW788388, using an experimental model of chronic Chagas’ heart disease (Ferreira et al., 2019). Treatment with the TGF-β receptor inhibitor improved several cardiac parameters, with reversion of heart fibrosis followed by a better heart function. However, the clinical use of inhibitors of TGF-β receptor kinase activity could induce some deleterious secondary effects (reviewed in Araújo-Jorge et al., 2012). Thus, here we also investigated the monoclonal antibody 1D11 during the experimental chronic phase of Chagas disease. 1D11 antibody has already been studied in other preclinical models; it was applied in a model of kidney fibrotic disease in which it was observed that mice receiving 1D11 from day 3 presented prevention of glomerular fibrosis even when started after the onset of proteinuria (Liang et al., 2016). A more recent work used 1D11 to demonstrate that aberrant activation of transforming growth factor-β (TGF-β) mobilizes mesenchymal/stromal stem cells in blood, which are recruited for the prostatic stromal hyperplasia (Wang et al., 2017). Hypertensive induced myocardial fibrosis was also studied under the effect of 1D11 *in vivo* and *in vitro* treatment (Wong et al., 2018), where it was shown that connective tissue growth factor (CTGF) expression is dependent on TGF-β signaling in a model of myocardial fibrosis. Another study also demonstrated the beneficial IP administration of 1D11 in a model of hepatic fibrosis (Ling et al., 2013). 1D11 administration was also effective to prevent radiation-induced lung injury (Anscher et al., 2006). These important findings corroborate the application of TGF-β neutralization in the complex cardiac fibrotic disorder observed by *T. cruzi* infection.

1D11 therapy ameliorates critical aspects of the disease. Therefore, TGF-β emerges as a candidate to treat the fibrosis deregulation associated with chronic Chagas’ heart disease and to improve patient prognosis. These data strongly reinforce that the treatment with antibodies could represent a new therapeutic strategy to address acute and chronic phases of Chagas disease that warrants further clinical exploration. We have conducted a series of studies investigating the role of TGF-β and its pathway inhibition. Here, we have again reinforced the importance of using TGF-β pathway inhibitors in Chagas chronic disease development. Thus, as a strategic step, pharmaceuticals worldwide should reinforce the development of humanized antibodies against TGF-β to become suitable for human tests. Finally, on-target anti-TGF-β therapies should be addressed in clinical trials in order to test its efficacy in patients presenting the cardiac disorder due to chronic Chagas disease.

## Data availability statement

The raw data supporting the conclusions of this article will be made available by the authors, without undue reservation.

## Ethics statement

The animal study was reviewed and approved by Institutional Committee for Animal Ethics of Fiocruz (CEUA/Fiocruz, Licenses LW10/14 and LW42-11).

## Author contributions

Conceptualization: SL, ES, MW. Formal analysis: RF, RA, GV-P, MM-B, NF, ES, JL-V, TA-J, MW. Funding acquisition: SL, JL-V, MW. Investigation: RF, RA, GV-P, MM-B, RB, ES, MW. Methodology: RF, RA, GV-P, MM-B, NF, ES, MW. Project administration: MW Resources: WD, JL-V. Supervision: ES, MW. Writing – original draft: RF, MW. Writing – review and editing: WD, ES, SL, JL-V, TA-J, SB. All authors contributed to the article and approved the submitted version.

## Funding

This work was supported by grants from MCW-Fundação Oswaldo Cruz (FIOCRUZ), JLV Fundação de Amparo à Pesquisa do Estado do Rio de Janeiro (FAPERJ), Temáticos/ process E-26/110.153/2013, and JLV-Conselho Nacional de Desenvolvimento Cientifico e Tecnologico (CNPq), DECIT negligenciadas process 403979/2012-9.

## Acknowledgments

The authors would like to thank the Laboratory Animals Breeding Center from Institute Oswaldo Cruz for technical support on animal care. The collaboration between the Brazilian and French teams was supported by an INSERM-FIOCRUZ collaborative program and the collaboration between the Brazilian and Genzyme teams was supported by the Genzyme-FIOCRUZ partnership developed under the leadership of Carlos Morel from the Health Technological Development Center (Centro de Desenvolvimento Tecnológico em Saúde -CDTS), Fundação Oswaldo Cruz, Rio de Janeiro, RJ, Brazil.

## Conflict of interest

The authors declare that the research was conducted in the absence of any commercial or financial relationships that could be construed as a potential conflict of interest.

The reviewer CC declared a shared affiliation with the authors RF, ES, WD, RA, and MW to the handling editor at the time of review.

## Publisher’s note

All claims expressed in this article are solely those of the authors and do not necessarily represent those of their affiliated organizations, or those of the publisher, the editors and the reviewers. Any product that may be evaluated in this article, or claim that may be made by its manufacturer, is not guaranteed or endorsed by the publisher.

## Supplementary material

Supplementary Figure 1 D11 administration affected parasitemia and mortality. Treatment was administered in different schemes: single dose at 3 dpi (1D11 d3), single dose at 5 dpi (1D11 d5), treatment starting at day 0+ three times a week (1D11 d0+ 3xw), starting at day 3+ once a week (1D11 d3+ 1xw) and starting at day 3+ three times a week (1D11 d3+ 3xw). Parasitemia was measured by direct counting of parasites in blood all along the acute phase **(A–E)**. Percent survival was monitored during the experiment until 30 dpi **(B–F)**. 1D11 treatment showed inhibition of parasitemia peak at 8 dpi in different schemes: 1D11 d5, 1D11 d0+ 1xw, 1D11 d3+ 1xw and 1D11 d0+ 3xw **(C)**. 1D11 treatment also showed a dose-dependent inhibition of parasitemia peak from 0.1 up to 10 mg/kg of 1D11 **(D)**. Data are means ± standard deviations from three independent experiments. Significant differences between infected mice treated or not with 1D11 are indicated by *p< 0.05 **p< 0.01, ***p< 0.001.
