# Supplementary figures and images for: In Chagas disease, transforming growth factor beta neutralization reduces Trypanosoma cruzi infection and improves cardiac performance
Source: Front Cell Infect Microbiol. 2022 Nov 30;12:1017040. doi: 10.3389/fcimb.2022.1017040 (PMC9748701; doi:10.3389/fcimb.2022.1017040)

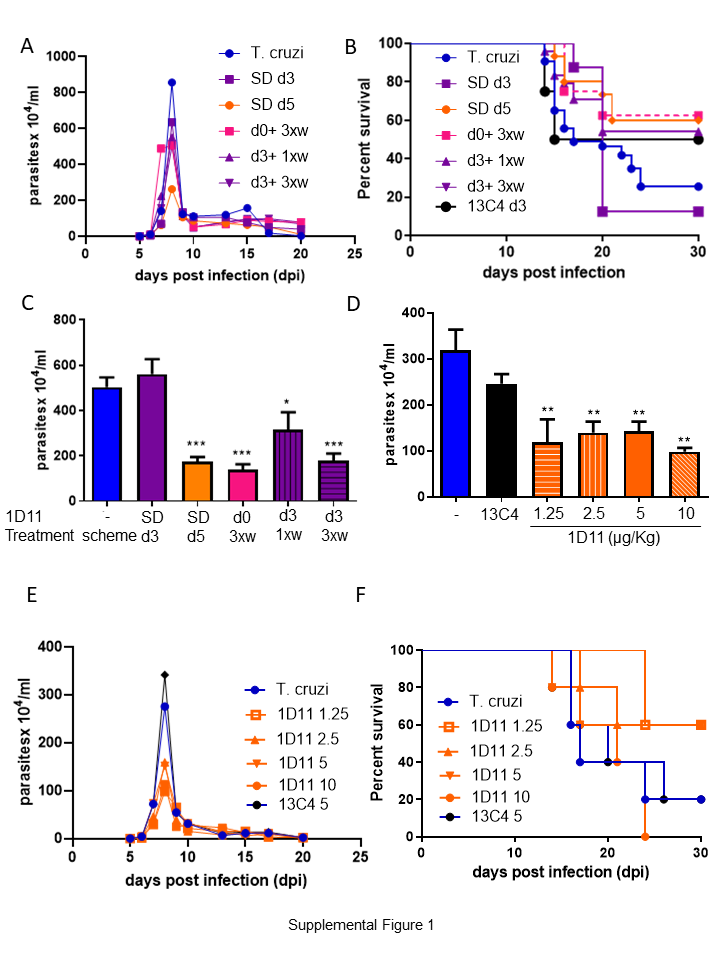

Supplement: Supplementary Figure 1 — D11 administration affected parasitemia and mortality. Treatment was administered in different schemes: single dose at 3 dpi (1D11 d3), single dose at 5 dpi (1D11 d5), treatment starting at day 0+ three times a week (1D11 d0+ 3xw), starting at day 3+ once a week (1D11 d3+ 1xw) and starting at day 3+ three times a week (1D11 d3+ 3xw). Parasitemia was measured by direct counting of parasites in blood all along the acute phase (A–E). Percent survival was monitored during the experiment until 30 dpi (B–F). 1D11 treatment showed inhibition of parasitemia peak at 8 dpi in different schemes: 1D11 d5, 1D11 d0+ 1xw, 1D11 d3+ 1xw and 1D11 d0+ 3xw (C). 1D11 treatment also showed a dose-dependent inhibition of parasitemia peak from 0.1 up to 10 mg/kg of 1D11 (D). Data are means ± standard deviations from three independent experiments. Significant differences between infected mice treated or not with 1D11 are indicated by *p< 0.05 **p< 0.01, ***p< 0.001. [file Image_1.tif]
